# Self-Compassion and Subjective Well-Being Mediate the Impact of Mindfulness on Balanced Time Perspective in Chinese College Students

**DOI:** 10.3389/fpsyg.2019.00367

**Published:** 2019-02-22

**Authors:** Jingjing Ge, Jun Wu, Kesheng Li, Yong Zheng

**Affiliations:** ^1^Faculty of Psychology, Southwest University, Chongqing, China; ^2^Guiyang University of Chinese Medicine, Guiyang, China; ^3^School of Marxism, Chongqing University of Science and Technology, Chongqing, China; ^4^College of National Culture and Cognitive Science of Guizhou Minzu University, Guiyang, China

**Keywords:** mindfulness, self-compassion, subjective well-being, balanced time perspective, mediation

## Abstract

Balanced time perspective is associated with optimal social functioning and provides psychological benefits in times of stress. Previous studies have found that mindfulness is positively associated with balanced time perspective and might promote it. However, the mechanism through which mindfulness affects balanced time perspective remains unexplored. The purpose of the present study was to investigate the mediating role of self-compassion and subjective well-being in the relationship between mindfulness and balanced time perspective. A total of 754 Chinese college students, aged 17–27 years, completed the Chinese versions of the Five-Facet Mindfulness Questionnaire, Self-Compassion Scale, Subjective Well-Being Scale, and Zimbardo Time Perspective Inventory. There were significant positive correlations between mindfulness, self-compassion, subjective well-being, and balanced time perspective. Structural equation modeling indicated that in addition to the direct influence of mindfulness on balanced time perspective, self-compassion and subjective well-being played a partial mediating role. On the basis of these findings, we conclude that mindfulness has an important positive influence on balanced time perspective, and highlights the crucial role of the self-compassion in cultivating a balanced time perspective. Limitations of the present study are also discussed.

## Introduction

Today’s society is fast-paced and highly competitive, and can lead individuals to feel pressured and develop psychological problems (Alsubaie et al., 2017; Littlewood et al., 2017). Psychological research has focused on how to maintain individuals’ physical and mental health, and reduce the adverse effects of modernization (Xinjian and Mengwei, 2013; Jiafei et al., 2014). Several studies have found that balanced time perspective (BTP) is beneficial for the individual not only to adapt to changing environments and pressure but also to maintain physical and mental health as well as optimal social functioning (Zhang et al., 2013; Sobol-Kwapinska and Jankowski, 2016). BTP can help individuals cope with life challenges, relieve stress, and achieve a balanced and happy life (Drake et al., 2008; Sobol-Kwapinska, 2016; Stolarski et al., 2016). These advantages of BTP make it the focus of positive psychology (Boniwell and Zimbardo, 2015), and studies are increasingly beginning to focus on BTP.

Balanced time perspective was first proposed by Zimbardo and Boyd (1999) in their study on the structure of time perspective (TP). TP has been viewed as the cognitive tendency of individuals to be time-oriented toward the past, present, or future. They defined five temporal frames to describe TP, as follows: Present Hedonistic, Present Fatalistic, Past Positive, Past Negative, and Future. These researchers further proposed the view of BTP. BTP was considered to be the most adaptive attitude toward temporal frames, and it refers to the ability to flexibly and effectively switch among different time perspectives depending on task features, situational considerations, and personal resources (Zimbardo and Boyd, 1999; Drake et al., 2008). Having BTP means that a person is good at adjusting himself when facing different situations, using the appropriate time perspective, and is able to “work hard when there is a mission to be accomplished, but play hard when the work is done” (Zimbardo, 2002). For example, for an office worker, having a future time perspective could improve work efficiency, and a present hedonistic time perspective could facilitate physical and mental relaxation on the weekend. Sometimes it is necessary to use past positive time perspective to develop self-confidence, and self-efficacy.

Stolarski and others considered that the deviation from the balanced time perspective (DBTP) could be regarded as an index of general temporal adaptation (Stolarski et al., 2015), while DBTP was opposite to the BTP. The lower the DBTP value was, the higher the BTP value would be (Stolarski et al., 2016). DBTP was negatively related to mindfulness, responsibility, life satisfaction, well-being, self-esteem and optimism (Stolarski et al., 2011; Sobol-Kwapinska, 2016).

Mindfulness involves consciously focusing on internal and external experiences in the present moment with a non-judgmental and accepting attitude (Brown and Ryan, 2003; Kabat-Zinn, 2003). Methods such as mindfulness meditation have been used to cultivate and enhance the level of individual mindfulness (Baer, 2003; Creswell et al., 2014; de Castro, 2015; Bianco et al., 2016). A large number of studies have shown that mindfulness meditation significantly reduces stress, improves physical and mental health, and produces satisfactory changes in life (Krygier et al., 2013; Creswell et al., 2014; de Jong et al., 2016; Lomas et al., 2017).

Some researchers have suggested that mindfulness may be an effective way to promote and maintain BTP (Drake et al., 2008; Stolarski et al., 2016). Cultivating BTP requires the individual’s awareness of his/her own time perspectives, and the ability to switch flexibly between different environments (Stolarski et al., 2015). Mindfulness emphasizes conscious awareness, focused attention on the present, and non-judgmental acceptance of everything in the present. Because of its influence on these attentional aspects, mindfulness may maintain and improve the individual’s attention to time perspectives. Stolarski et al. (2016) showed that mindfulness had a significant positive correlation with BTP, and mindfulness predicted BTP. Thus, mindfulness meditation may be an effective way to increase individuals’ BTP. However, the process by which mindfulness affects BTP is unclear and research on this topic has been scarce.

### Mindfulness, Self-Compassion, and BTP

Self-compassion has been defined as being kind toward oneself when facing difficulties and failure (Neff, 2003a; Neff et al., 2008). Individuals with high levels of self-compassion are less likely to experience more negative emotions when faced with negative life events (Neff and Vonk, 2009). They have a balanced view of suffering and are more objective in understanding negative life events. These individuals also tend to have more positive coping styles when encountering suffering (Leary et al., 2007). A growing number of studies have shown that self-compassion can promote resilience by buffering against and reducing the adverse effects of negative life events (Wong and Mak, 2013; Heath et al., 2017), and self-compassion is an important factor protecting in psychological health (Birnie et al., 2010; Neff and Germer, 2013). Higher levels of self-compassion are associated with higher levels of emotional intelligence and social connection, and lower levels of self-criticism, depression, anxiety, rumination, and thought suppression (Van Dam et al., 2011; Hitchcock et al., 2012; Ferguson et al., 2014; Neff and Faso, 2014). Studies have shown that mindfulness training can effectively enhance individuals’ cognitive function and self-compassion (Kuyken et al., 2010). Those with high levels of mindfulness also show high levels of self-compassion, and this positive correlation has been confirmed by several studies (Shapiro et al., 2005; Nedeljkovic et al., 2012; Duarte and Pinto-Gouveia, 2017). In recent years, research has identified self-compassion as an important factor in mindfulness training interventions (Birnie et al., 2010; Kuyken et al., 2010; Baer et al., 2012; Campos et al., 2016). In the intention, attention, and attitude (IAA) mindfulness model, attitudes are important (Shapiro et al., 2006), and self-compassion reflects attitudes toward oneself. Specific to the influence of mindfulness on TP, we believe it is possible that self - compassion is an important predictor variable, and it may be an important and critical element. Therefore, we think that self-compassion may play a mediating role in the relationship between mindfulness and BTP.

### Mindfulness, Self-Compassion, and Subjective Well-Being

Subjective well-being (SWB) is an individual’s cognitive and emotional evaluation of his/her own life. It is generally considered to be composed of three factors: life satisfaction, positive emotion, and negative emotion (Diener and Suh, 2000; Feng et al., 2012). Life satisfaction refers to an individuals’ cognitive assessment of life quality, while positive emotions and negative emotions refer to an individuals’ subjective emotional experience of life quality (Diener and Suh, 2000). Mindfulness enhances emotional regulation and coordination of cognition (Ortner et al., 2007; Friese et al., 2012; Short et al., 2016; Guendelman et al., 2017), which can effectively improve individuals’ SWB (Schoormans and Nyklicek, 2011; Chang et al., 2015). In addition, some studies have shown that self-compassion is also positively correlated with well-being (Bluth and Blanton, 2014, 2015). Individuals high in self-compassion tend to face their own shortcomings with a friendly and warm attitude. This attitude may affect the individual’s cognitive and emotional state, increase the individual’s positive experience and it may reduce the negative emotional experience. This may subsequently improve the individual’s SWB. Hollis-Walker and Colosimo (2011) found that mindfulness and self-compassion can predict SWB, and the relationship between mindfulness and SWB is mediated by self-compassion. Therefore, this study speculated that self-compassion plays an intermediary role between mindfulness and SWB.

### Self-Compassion, SWB, and BTP

Individuals with higher SWB have more positive emotions, less negative emotions, and higher life satisfaction. According to the Broaden-and-Build Theory of Positive Emotions, positive emotions can broaden the range of cognition improve the flexibility of cognition and thinking, and promote creative problem solving (Fredrickson, 1998, 2001; Mikulincer and Sheffi, 2000). Based on the Broaden-and-Build Theory of Positive Emotions, we speculate that when positive cognitive and emotional experiences are high, and negative cognitive and emotional experiences are low, individuals may perceive people and things in their surroundings from a more positive perspective. This may help individuals to maintain good interpersonal relationships, and they may in turn be able to gain more social support. A happy state of mind can also play a protective role and encourage individuals to be more active, as well as promote individuals to treat life and time with a more flexible perspective, conducive to developing and maintaining a BTP. Thus we speculate that SWB may predict BTP. In addition, several studies have documented a positive correlation between SWB and BTP, and BTP can predict SWB. However, these researches were designed as cross-sectional studies that could not determine the true causal relationship as longitudinal studies. Moreover, a study performed by Stolarski et al. (2016)discovered that BTP mediated the relationship between mindfulness and life satisfaction, but proved that the reverse model was valid at the same time, which consequently indicated the possibility of BTP predicted SWB, and vice versa. Therefore, it was speculated in this study that SWB could also predict BTP.

Self-compassion improves life satisfaction and positive emotions, reduces negative emotions, and promotes SWB (Baer et al., 2012). Therefore, this study assumes that SWB plays an intermediary role between self-compassion and BTP.

### The Present Study

The IAA mindfulness model (Shapiro et al., 2006) proposes the mechanism by which mindfulness may work. Per this model, mindfulness consists of three components: attention (self-regulation and current moment orientation), intention (purpose) and attitude (friendliness, non-judgment and self-compassion), these three components lead to increases in four areas: self-regulation, values clarification, cognitive and emotional and behavioral flexibility, and exposure. These variables can be seen as potential mechanisms for other outcomes, such as stress reduction. Specific to the influence of mindfulness on TP, based on the close relationship between TP and attention, attitude, cognition and emotion, this study combines mindfulness (relating to attention), self-compassion (relating to attitudes toward oneself), and SWB (relating to the cognitive and emotional evaluation of one’s own quality of life) to better understand the mechanism between BTP and mindfulness.

The present study used a chain-based multiple mediation model to examine the relationship between mindfulness and BTP while considering self-compassion and SWB as mediating variables. We propose the following three hypotheses:

Hypothesis 1: self-compassion plays a mediating role in the relationship between mindfulness and BTP, namely, the existence of a “mindfulness → self-compassion → BTP” path.
Hypothesis 2: SWB plays a mediating role in the relationship between mindfulness and BTP, namely, the existence of a “mindfulness → SWB → BTP” path.
Hypothesis 3: mindfulness can significantly predict BTP through the serial mediating role of self-compassion and SWB, namely, the existence of a “mindfulness → self-compassion → SWB → BTP” path.

**FIGURE 1:**
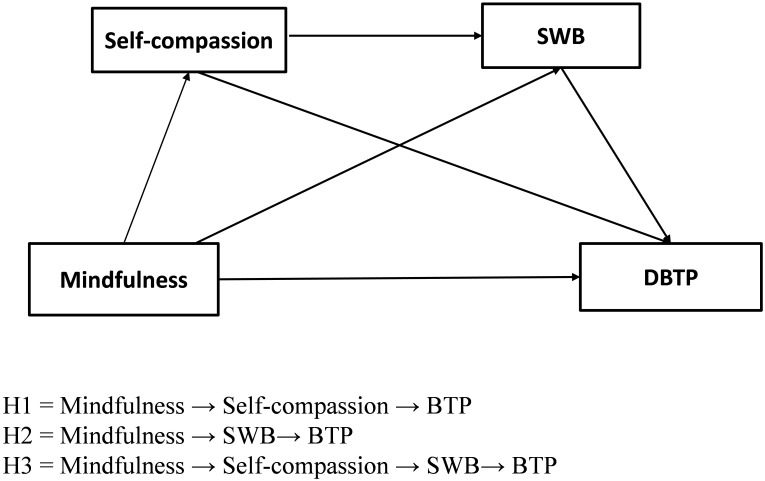
The hypothesis model of the relationship between mindfulness and BTP. DBTP, deviation from the balanced time.

To test these hypotheses, we constructed a hypothetical multiple mediation model to investigate whether mindfulness could promote BTP through self-compassion and SWB (Figure 1).

## Materials and Methods

### Participants

This study gathered convenience samples from five Chinese universities in Guizhou province and Chongqing municipality. Participants in the current study were 754 college students; 42.71% (*n* = 322) were male and 57.29% (*n* = 432) female. Participants’ age ranged from 17 to 27 years, with a mean age of 20.62 years (*SD* = 1.70). The number of participants in the first grade was 226, followed by second grade (239), third grade (187), and fourth grade (102).

We used the class collective measured approach to obtain the data, and recycled the questionnaires immediately after each survey was completed. Written informed consent was obtained from each participant before the commencement of the study.

### Measures

#### Mindfulness

Mindfulness levels were measured using the Five-Facet Mindfulness Questionnaire (FFMQ) (Baer et al., 2006). Deng et al. (2011) developed the Chinese version of the FFMQ, which was used in this study. This 39-item scale consists of five subscales, namely, observing, describing, non-judgment of inner experience, non-reactivity to inner experience, and acting with awareness. Two example questions are, “When I take a shower or bath, I stay alert to the sensations of water on my body” and “I can easily put my beliefs, opinions, and expectations into words.” The Chinese FFMQ is scored on a 5-point Likert scale. A total mindfulness score as well as subscale scores were calculated, with higher scores indicating higher levels of mindfulness. The Chinese FFMQ has shown acceptable psychometric properties with a sample of Chinese university students (Deng et al., 2011). For our analyses, we used a composite score as well as factor scores, and the reliability coefficient of the whole scale was 0.706 in this study.

#### Self-Compassion

Self-compassion was measured using the Chinese version of the Self-Compassion Scale, originally developed by Neff (2003b). The scale and its Chinese translation have been used in cross-cultural research and good reliability and validity were confirmed (Neff, 2003b; Neff et al., 2008; Jian et al., 2011). It contains 26 items and three factors: self-kindness, common humanity, and mindfulness. Two example questions are, “I try to see my failings as part of the human condition” and “When I’m feeling down I tend to obsess and fixate on everything that’s wrong.” Items are rated using a 5-point Likert scale from 1 (almost never) to 5 (almost always). In the current sample, the reliability coefficient of the whole scale was 0.83, and each subscale was 0.63, 0.65, and 0.63, respectively.

#### Subjective Well-Being (SWB)

Subjective well-being was measured using the Chinese version of the SWB Scale, originally developed by Diener et al. (2000). The scale and its Chinese translation have been used in cross-cultural research and good reliability and validity were confirmed (Diener and Suh, 2000; Diener et al., 2000; Feng et al., 2012). This scale contains a total of 19 items, scored on a 7-point Likert scale, divided in three factors: life satisfaction, positive affection, and negative affection. One example questions is, “In most ways, my life is close to my ideal.” The life satisfaction subscale consists of five items measuring global cognitive judgments of one’s life, and the positive and negative affection subscales include six and eight items, respectively. In the current sample, the reliability coefficient of each subscale was 0.71, 0.78, and 0.79, respectively.

#### Zimbardo Time Perspective Inventory

The Zimbardo Time Perspective Inventory (Chinese version) was used to measure time perspective (Zimbardo and Boyd, 1999). This instrument has shown good reliability and validity cross-culturally (Sircova et al., 2014). It comprises a total of 56 items rated on a 5-point Likert scale and divided into five subscales: Past Negative (PN), Past Positive (PP), Present Fatalistic (PF), Present Hedonistic (PH), and Future (F). Two example questions are, “It gives me pleasure to think about my past” and “I believe that a person’s day should be planned ahead each morning.” In the current sample, the reliability coefficient of each subscale was 0.75, 0.73, 0.65, 0.69, and 0.74, respectively.

#### Balanced Time Perspective (BTP)

Zimbardo and Boyd (1999) presented the view of BTP, using the deviation from a balanced time perspective (DBTP) to measure the individual’s BTP level (Stolarski et al., 2011). DBTP is based on Zimbardo Time Perspective Inventory scores, and indicates how close or far an individual is from having a BTP (Zhang et al., 2013). Among the several calculation methods of BTP, the DBTP is regarded as the most appropriate method to measure BTP (Stolarski et al., 2011; Zhang et al., 2013). DBTP is a continuous indicator of the degree to which an individual’s empirical time perspective (*e*TP) profile approximates the optimal time perspective (*o*TP) profile.

$$\text{DBTP}=\sqrt{{\left(o\text{PN}-e\text{PN}\right)}^{2}+{\left(o\text{PP}-e\text{PP}\right)}^{2}+{\left(o\text{PF}-e\text{PF}\right)}^{2}+{\left(o\text{PH}-e\text{PH}\right)}^{2}+{\left(o\text{F}-e\text{F}\right)}^{2}}$$

Where (*o*F -*e*F) is (optimal Future) – (an individual’s empirical Future); Repeat the process for each time perspective dimension. Zimbardo and Boyd (2008) proposed optimal scores for all the time perspective dimensions based on their collective cross-cultural dataset. The negative correlation between DBTP and certain concept indicated that BTP had a positive correlation with the concept (Vowinckel et al., 2017). There was a negative correlation between DBTP and BTP, the lower the DBTP is, the higher the BTP.

### Procedures

This study was approved by the ethics committee of the Faculty of Psychology at the Southwest University, China. Written informed consent was obtained from each participant before the commencement of the study. The researcher instructed the participants for completing the surveys in the classroom environment. Participants completed surveys consisting of the Five-Facet Mindfulness Questionnaire, Self-Compassion Scale, SWB Scale, and Zimbardo Time Perspective Inventory. The researcher assured the participants of the confidentiality of their answers. It took approximately 20 min for the students to complete the surveys.

### Statistical Analyses

All data in this study were analyzed using SPSS 23.0 and Amos 23.0. Firstly, we examined the descriptive statistics and correlations of the study variables using SPSS, and the mean, standard deviation, and correlation of the sample and related variables were obtained. Subsequently, we examined the pattern of relationships in our theoretical model through a path analysis using AMOS. The path analysis was used to test the direct and indirect relationships among variables, which can provide estimates of the magnitude and significance of the causal connections hypothesized between variables. The bias-corrected percentile bootstrap method with 5000 bootstrap samples was selected to confirm the significance of the mediating effects of self-compassion and SWB on the link between mindfulness and BTP. This method is included as an option in AMOS and it produces the most accurate confidence limits with the largest power for detecting mediation effects (WEN and YE, 2014).

## Results

### Common Method Bias Control

Data in this study were generated through self-report, and common method bias owing to common method variance in the measurement may thus exist (Xiong et al., 2012). This study used process control to regulate common method variance, including (1) use of scales with relatively high reliability and validity as measurement tools; (2) anonymized data, people did not include their names on the questionnaires; and (3) keeping participant information strictly confidential.

After completion of data collection, the Harman single factor test was used to test for common method bias (Hao and Lirong, 2004). The results showed that the first factor only accounted for 9.631% variance, which was much lower than the critical standard of 40%, suggesting that common method bias was not obvious (Li and Li, 2015).

### Descriptive Statistics

The mean, standard deviation, and correlation coefficient of each variable were calculated and results are shown in Table 1. Mindfulness was significant positive correlations (*p* < 0.01) with self-compassion, SWB, past positive time perspective, and future time perspective. Self-compassion was significant positive correlations (*p* < 0.01) with mindfulness, SWB, past positive time perspective, and future time perspective. DBTP was negatively correlated with mindfulness, self-compassion, SWB, past positive time perspective, and future time perspective (*p* < 0.01), and positively correlated with past negative and present fatalistic time perspectives (*p* < 0.01).

**Table 1:** Mean, standard deviation, and association among study measures.

Variable	*M*	*SD*	1	2	3	4	5	6	7	8	9	10
(1) Mindfulness	3.033	0.278	–									
(2) Self-compassion	3.131	0.420	0.446^**^	–								
(3) SWB	4.205	0.633	0.331^**^	0.394^**^	–							
(4) Past negative	3.028	0.588	-0.331^**^	-0.467^**^	-0.371^**^	–						
(5) Past positive	3.687	0.523	0.227^**^	0.152^**^	0.265^**^	-0.142^**^	–					
(6) Present fatalistic	2.658	0.555	-0.299^**^	-0.312^**^	-0.258^**^	0.477^**^	-0.160^**^	–				
(7) Present hedonistic	3.208	0.455	-0.010	-0.012	0.056	0.213^**^	0.225^**^	0.315^**^	–			
(8) Future-oriented	3.365	0.443	0.347^**^	0.260^**^	0.197^**^	-0.095^**^	0.303^**^	-0.286^**^	0.054	–		
(9) DBTP	2.269	0.631	-0.418^**^	-0.436^**^	-0.423^**^	0.683^**^	-0.593^**^	0.682^**^	-0.086^*^	-0.479^**^	–	
(10) Age	20.62	1.699	0.075^*^	0.071	0.016	-0.016	-0.047	-0.005	-0.043	-0.019	0.005	–
(11) Sex	1.57	0.495	0.016	-0.077^*^	-0.004	-0.036	0.007	-0.123^**^	-0.106^**^	0.100^**^	-0.067	-0.060

*N = 754; ^∗^p < 0.05, ^∗∗^P < 0.01, ^∗∗∗^p < 0.001.*

### Testing the Multiple Mediation Model of Mindfulness Influencing BTP

**FIGURE 2:**
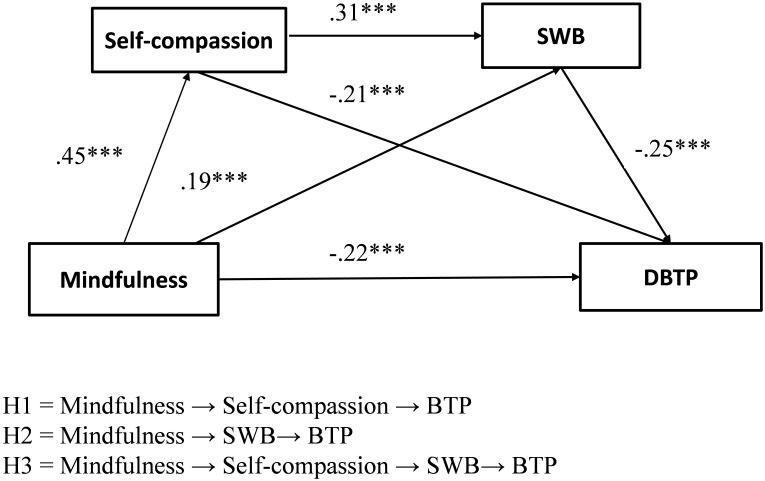
The multiple mediator model of mindfulness and BTP. DBTP, deviation from the balanced time perspective, based on the ZTPI scores was applied as an indicator of BTP. Lower DBTP scores show a higher level of BTP. ^∗^*p* < 0.05, ^∗∗^*p* < 0.01, ^∗∗∗^*p* < 0.001.

Based on the proposed mediating model shown in Figure 1 and the correlations of all of the measures in Table 1, a path analysis was conducted in AMOS, to test the total effect of mindfulness on BTP and the three specific mediating effects. The standardized estimated path coefficients for these effects have been shown in Figure 2.

Firstly, in the mediation model, after controlling for the effects of gender and age, mindfulness significantly predicted BTP (β = -0.51, standardized β = -0.22, *p* < 0.001) and SWB (β = 0.44, standardized β = 0.19, *p* < 0.001); Self-compassion significantly predicted SWB (β = 0.46, standardized β = 0.31, *p* < 0.001) and BTP (β = -0.36, standardized β = -0.24, *p* < 0.001). SWB significantly predicted BTP (β = -0.25, standardized β = -0.25, *p* < 0.001).

Secondly, as shown in Table 2, the assessment of the indirect effects in this multiple mediator model suggested a significant indirect serial mediated effect of self-compassion and SWB (95% CI = [-0.12, -0.05]), Additionally, analyses revealed two separate indirect effects through self-compassion (95% CI = [-0.35, -0.17]) and SWB (95% CI = [-0.17, -0.07]) in the relationship between mindfulness and BTP. These results suggested that self-compassion and SWB partly mediated the effect of mindfulness on BTP.

**Table 2:** Indirect effects of mindfulness on BTP.

Paths of indirect effect	Effect size ( standardized β )	95% CI
Mindfulness → Self-compassion → DBTP	0.45 × (-0.24) = (-0.11)^∗∗∗^	[-0.35, -0.17]
Mindfulness → SWB → DBTP	0.19 × (-0.25) = (-0.05)^∗∗∗^	[-0.17, -0.07]
Mindfulness → Self-compassion → SWB → DBTP	0.45 × 0.31 × (-0.25) = (-0.03)^∗∗∗^	[-0.12, -0.05]

*N = 754. The path coefficient in the model is standardized coefficient (Standardized β). ^∗∗^p < 0.01, ^∗∗∗^p < 0.001.*

## Discussion

Based on the IAA mindfulness theoretical model, the current study examined mediating models on the relationships among mindfulness, self-compassion, SWB, and BTP in Chinese college students. The present findings suggest that self-compassion and SWB may partly mediate the effect of mindfulness on BTP as serial and parallel inductors. Correlation analysis shows that there is a significant positive correlation between mindfulness, self-compassion, SWB and BTP. And mindfulness can positively predict self-compassion, SWB and BTP. The presented results are in line with previous researches (Baer et al., 2012; Bluth and Blanton, 2014; Stolarski et al., 2016).

This study also found that mindfulness has an indirect effect on BTP through the mediation of self-compassion. Individuals with low self-compassion have cognitive biases based on past experiences, and often have negative self-perceptions. However, mindfulness is a balanced consciousness that emphasizes non-judgmental awareness and acceptance. This way of treating internal and external experiences helps to reduce self-criticism and redundancy, avoiding the excessive influence of cognitive biases (Jain et al., 2007; Samaie and Farahani, 2011; Hitchcock et al., 2012). Self-compassion is a positive personal resource (Leary et al., 2007), it can buffer the influence of negative emotions and thoughts on BTP, thereby improving the level of BTP. Namely, those with high levels of self-compassion tend to have more positive attitudes and more positive cognitive evaluations of themselves. These individuals experience more positive emotions, high self-worth (Neff and Vonk, 2009), and self-efficacy (Iskender, 2009), so they have higher BTP. Individuals with low self-compassion tend to have negative attitudes and worse cognitive evaluations on themselves. These individuals experience low self-worth (Neff and Vonk, 2009), lack self-confidence, and think that they are not as good as others. As such, these individuals often have a low sense of self-efficacy, their BTP is lower. Therefore, self-compassion is an intermediary between mindfulness and BTP.

In the chain mediating pathway between mindfulness and BTP, SWB plays a mediating role between self-compassion and BTP. SWB is an important psychological characteristic within the field of positive psychology. According to the Broaden-and-Build Theory of Positive Emotions (Fredrickson, 1998, 2001), SWB has the function of expanding and enhancing cognition and thinking abilities. Individuals with high SWB hold relatively positive cognitive appraisal of their quality of life and experience of subjective emotion. The balance and stability of emotion as well as the flexibility and pioneering characteristics of cognitive will conducive to the maintenance and development of individual BTP. Self-compassion can adjust the psychological impact of life stress on individuals, increase the ability to control negative emotions, and make their emotional experience and subjective evaluation more positive, so as to maintain a high SWB. Self-compassion can improve individuals’ cognition and emotional experience of the quality of life, and indirectly affects BTP through the mediating effect of SWB.

Structural equation model analysis shows that self-compassion and SWB are partially mediated between mindfulness and BTP. The direct effect of mindfulness on BTP is significant, and higher than the total indirect effect, indicating that the positive impact of mindfulness on BTP is very important. Firstly, mindfulness implies maintaining a conscious awareness of the present moment (Drake et al., 2008; Stolarski et al., 2016), and can be interpreted as the self-regulation of attention, involving flexibility, sustainability, and conversion of attention (Baer, 2003; Bishop et al., 2004; Castelli and Tesio, 2016; Crescentini et al., 2016; Tang et al., 2017). Mindfulness may enhance BTP by increasing awareness and attention. On the one hand, it makes it easier for individuals to build awareness of a time perspective. On the other hand, individuals can be more sensitive to the needs of the current external environment and tasks, and can flexibly adjust the time perception according to the needs of tasks. Secondly, some studies are now focusing on the relationship between mindfulness and time experience. Mindfulness has been found to affect time perception, and even just about 10 min of mindfulness meditation can slow down time perception (Kramer et al., 2013). When mindfulness is seen as a personality trait, it was found that mindfulness was associated with a series of time-related personality traits, such as daily time planning and decision making, self-control of time, higher future time perspective, and lower past negative time perspective (Wittmann et al., 2014). Thirdly, people are usually in the “doing mode of mind,” purpose-oriented and emphasizing evaluation and change. Mindfulness emphasizes awareness and acceptance, focuses on the present experience, and is a “being mode of mind” (Brown and Ryan, 2003; Williams, 2010; Dorjee, 2016), which can change individuals’ time experience, time consciousness, and time perception (Otten et al., 2015; Wittmann et al., 2015; Schötz et al., 2016), and thus influence BTP. Finally, the direct effect of mindfulness on BTP also suggests that mindfulness may enhance BTP through other paths. In addition to self-compassion and SWB, there may be other mediating variables. Future research should focus on finding other mediating variables and pathways so as to reveal more clearly and systematically the inner mechanism of the positive effect of mindfulness on BTP.

Finally, studies have shown that both mindfulness and self-compassion positively predict BTP. BTP is believed to be related to the individual’s optimal social function, and is also the central concept of TP therapy (Sword et al., 2014). TP therapy is a simple and effective method, which is not only applicable to depression, post-traumatic stress disorder (PTSD), domestic violence, traffic accident trauma, but also for people’s daily life adjustments (e.g., study stress, career burnout, procrastination, and so on). Mindfulness, self-compassion, and TP are closely related to mental health functioning, behavior, emotions, and cognitive decision-making. Fortunately, mindfulness can be learned through mindfulness training and meditation. Self-compassion can be learned through self-compassion training and loving-kindness meditation. We can integrate the content of mindfulness and self-compassion training into TP therapy and use mindfulness and self-compassion training to adjust and improve TP, so as to provide basis for effective.

### Limitations

A few limitations of the present study merit consideration. Firstly, the results relied entirely upon self-report data, and this approach has potential problems, such as inaccurate responses from participants. Secondly, as the current study was conducted with a sample of college students in China, whether our findings could be generalized to different age groups and cultures remains to be determined. Thirdly, due to the convenient sampling method, the representativeness of the sample is relatively poor, and the results of the survey may not apply to the general population. Fourthly, we used self-compassion and BTP as the mediators in a model of mindfulness affecting SWB, and path analysis was also established. It was detected that SWB could predict BTP, and vice versa, which were similar to the results provided by Stolarski et al. (2016). As revealed in the study by Stolarski et al. (2016), BTP mediated the relationship between mindfulness and life satisfaction, but a test for reverse causation proved that the reverse model was valid. Consequently it indicated that SWB and BTP may interact with each other. Since this study has a cross-sectional design, it was not possible to make causal inferences. Therefore, the explanation proposed here should be treated with appropriate caution. Future research should focus on longitudinal investigations.

## Conclusion

This study may be the first to explore the mediating role of self-compassion in the relationship between mindfulness and BTP. This study expands upon existing knowledge regarding the relationship between mindfulness and BTP, and its findings are novel and insightful, both theoretically and practically.

Our findings suggest that mindfulness positively predicts BTP. There were significant positive correlations between mindfulness, self-compassion, SWB, and BTP. Structural equation modeling indicated that self-compassion and SWB played a partial mediating role in addition to the direct influence of mindfulness on BTP. On the basis of these findings, we conclude that mindfulness has an important positive influence on BTP. Students with higher levels of mindfulness may have higher self-compassion and SWB, thus having higher BTP. The present study offers an important foundation for future work.

## Author Contributions

JG participated in the design, data collection, data analysis, data interpretation, and drafting the early version of the article. JW and KL participated in the design and data collection. YZ participated in the design, data collection, and revising the article critically for better intrinsic logicality.

## Conflict of Interest Statement

The authors declare that the research was conducted in the absence of any commercial or financial relationships that could be construed as a potential conflict of interest.
